# Water, sanitation, and hygiene and nutritional status of children under-5 years born to young mothers in Ghana: a spatial inequality analysis

**DOI:** 10.3389/fnut.2026.1897491

**Published:** 2026-09-03

**Authors:** Deda Ogum, Yula Salifu, Abdul Razak Abubakari, Gilbert Abotisem Abiiro, Torjim Salifu, Jawahir Mohammed Abukari, Joseph Lasong

**Affiliations:** 1 Department of Population, Family and Reproductive Health, School of Public Health, University of Ghana, Accra, Ghana; 2 Department of Population and Reproductive Health, School of Public Health, University for Development Studies, Tamale, Ghana; 3 Department of Health Policy, Management and Economics, School of Public Health, University for Development Studies, Tamale, Ghana

**Keywords:** Ghana, socioeconomic inequality, spatial analysis, stunting, underweight, wash, wasting, young mothers

## Abstract

**Background:**

Malnutrition among children under five remains a major public health challenge in sub-Saharan Africa, with those born to young mothers being particularly vulnerable. Evidence on the role of water, sanitation, and hygiene (WASH) in this subgroup is limited in Ghana. We estimated the association between WASH and childhood malnutrition, and characterized its spatial and socioeconomic distribution, in young mother–child pairs in Ghana.

**Methods:**

We analyzed 620 child–mother pairs (mothers aged 15–24 years) from the 2022 Ghana Demographic and Health Survey. Outcomes were stunting, wasting, and underweight, defined using WHO growth standards (< −2 SD). Survey-weighted multilevel logistic regression examined WASH–nutrition associations. Spatial patterns were assessed using Global Moran’s I, and LISA and Getis-Ord G*. Socioeconomic inequality was measured via concentration indices, the Theil T index, Lorenz curves, and difference/relative indices. Population attributable fractions (PAF) and risks (PAR) were estimated for binary WASH exposures.

**Results:**

Stunting prevalence was 18.8% (95% CI 15.5–22.6), wasting 8.7% (6.2–11.9), and underweight 15.6% (12.4–19.4). Volta Region recorded the highest observed weighted prevalence for all three outcomes (stunting 32.0%, wasting 16.8%, underweight 39.4%), but these estimates were based on a small subsample (unweighted *n* = 20). Global spatial autocorrelation was not statistically significant for any outcome (all *p* ≥ 0.296). LISA identified Oti as a possible hotspot for underweight (*p* = 0.039), and Central and Bono as cold spots for stunting (*p* = 0.047) and wasting (*p* = 0.018). Improved water access showed the largest protective population-level association (PAF: stunting −36 to −41%; underweight −37%), interpreted as a population-level attributable-burden estimate under a cross-sectional design. Concentration indices were negative for all outcomes, indicating disproportionate burden among poorer and less-educated households, reaching statistical significance for underweight. WASH covariates were not significantly associated with any outcome in fully adjusted models. Multilevel models showed high apparent discrimination (AUC 0.859–0.868).

**Conclusion:**

Malnutrition among children of young mothers in Ghana is unevenly distributed and concentrated among socioeconomically disadvantaged populations. Equitable WASH services, alongside targeted nutrition programming, may help reduce nutritional inequalities in support of SDG 2 and SDG 6.

## Introduction

Children under five years constitute one of the most biologically and socially vulnerable population groups globally. During this critical period of rapid growth and development, children are highly susceptible to infectious diseases, malnutrition, environmental contaminants, and inadequate caregiving practices (1, 2). Malnutrition in this age group, particularly stunting, wasting, and underweight, adversely affects physical growth, cognitive development, immune function, and long-term human capital formation (1, 3).

Despite decades of policy interventions, the global burden of child malnutrition remains substantial. In 2023, approximately 4.8 million children died before reaching their fifth birthday, with nearly half of these deaths linked to undernutrition (4). Sub-Saharan Africa continues to bear a disproportionate burden (4). A pooled analysis of Demographic and Health Survey (DHS) data from 31 sub-Saharan African countries reported prevalences of 32.7% for stunting, 9.9% for wasting, and 21.4% for underweight, with marked regional variation (5). West Africa remains particularly affected due to persistent poverty, food insecurity, inadequate healthcare access, and limited water, sanitation, and hygiene (WASH) infrastructure (5, 6).

Ghana presents a mixed nutritional landscape. The 2022 Ghana Demographic and Health Survey (GDHS) reported that 17% of under-five children were stunted, an improvement from 28% in 2008 (7). Despite this progress, chronic malnutrition remains a critical public health concern with far-reaching developmental consequences. Children in households lacking access to basic hygiene services face substantially higher odds of growth faltering (8). Cumulative stunting produces largely irreversible deficits in cognitive development, educational attainment and adult economic productivity, while underweight reflects acute nutrient depletion that heightens vulnerability to infectious disease and premature mortality (1, 3, 8). The coexisting burden of stunting and underweight therefore signals not merely dietary inadequacy but the structural convergence of WASH deficits, entrenched poverty and constrained maternal agency. Substantial geographic inequalities persist: the northern regions of Ghana (Northern, North East, Savannah, and Upper East) consistently record higher levels of child malnutrition than southern regions, owing to longstanding disparities in poverty, healthcare access, food systems, and WASH infrastructure (7, 9). These regions, together with the Volta Belt, experience acute water insecurity including heavy dependence on unprotected surface sources, pronounced dry-season shortages, and among the highest rates of open defecation nationally (10). These regional differences highlight the need for spatially disaggregated analyses to identify vulnerable populations and guide targeted interventions.

The relationship between WASH conditions and child nutritional status is well established. The UNICEF conceptual framework identifies inadequate water, sanitation, and hygiene as key underlying determinants of undernutrition operating through infectious-disease pathways (11). Poor access to safe drinking water, unimproved sanitation, and inadequate hygiene increase exposure to diarrhoeal diseases, intestinal parasites, and environmental enteric dysfunction, all of which impair nutrient absorption and compromise growth (12–15). Mechanistically, unsafe water and unimproved sanitation increase the enteric infection burden and precipitate environmental enteropathy, a subclinical inflammatory condition characterized by villous blunting, crypt hyperplasia, reduced mucosal absorptive surface area and systemic microbial translocation (16), impairing absorption of protein, energy, zinc, iron and vitamin A and establishing a self-reinforcing cycle of infection, nutrient depletion and further growth faltering (12–14, 16).

Evidence from low- and middle-income countries consistently supports these pathways. Fink and colleagues (17), using pooled DHS data, found improved water and sanitation associated with lower child morbidity and better nutrition. Similar associations between inadequate sanitation and stunting have been reported in Ethiopia, Indonesia, India, and Benin (18–21).

Children born to young mothers may face additional vulnerabilities. In this study, young mothers refer to women aged 15–24 years, including adolescents (15–19) and young adults (20–24). Young mothers often experience overlapping biological, socioeconomic and structural disadvantages (22). Physiological immaturity among adolescent mothers increases the risk of low birth weight and preterm delivery (22). Young mothers are also more likely to experience poverty, limited education, reduced autonomy, and restricted access to healthcare and WASH resources (23, 24). Critically, they frequently lack household decision-making authority, which restricts their ability to direct resources toward safe water procurement, hygienic food preparation and optimal infant feeding (25). The physical burden of water collection, disproportionately borne by women and girls, further constrains the time available for exclusive breastfeeding and appropriate complementary feeding (25).

### Hypothesis of differential WASH vulnerability

We advance the hypothesis that the association between household WASH conditions and child nutritional status is stronger among children of young mothers than among children of older mothers, for three interlocking reasons. First, exposure differs: young mothers are over-represented in the poorest quintiles and in rural and peri-urban households where unimproved water and shared sanitation predominate (7, 23, 24). Second, the capacity to buffer that exposure differs: translating WASH access into safe practice requires household authority over expenditure, time and labour, which young mothers, typically residing in households headed by older adults, often lack (25, 26). Third, the biological susceptibility of the exposed child differs: children of young mothers are more likely to be born preterm or of low birth weight and weaned earlier, and are more vulnerable to the growth consequences of environmental enteropathy (16, 22, 27, 28). If this hypothesis holds, maternal age is an effect modifier of the WASH–nutrition relationship rather than a confounder. This reasoning motivates restricting the analytic sample to children of mothers aged 15–24 years rather than adjusting for maternal age in a pooled sample. We note explicitly that a formal test of effect modification would require the pooled sample with a maternal-age × WASH interaction term, which the present design does not estimate.

In Ghana, early childbearing is associated with lower maternal education, reduced healthcare utilization and household poverty (7, 23). Empirical evidence examining how WASH conditions influence the nutritional status of under-five children born specifically to young mothers in Ghana remains limited, and few studies have explored the spatial distribution and socioeconomic inequalities of malnutrition within this subgroup using advanced spatial approaches (29–32).

### Study objectives

The study addressed five objectives, each expressed as a question: (1) how common are stunting, wasting and underweight among under-five children of young mothers, and does the burden vary across Ghana’s 16 regions (2) is the regional variation spatially (3) how much of the burden is attributable to WASH deficits at population level (4) how is the burden distributed across socioeconomic strata and what drives that distribution (5) do WASH conditions predict individual risk once composition and context are controlled.

## Methods

### Study design and data source

This study employed a cross-sectional analytical design using secondary data from the 2022 Ghana Demographic and Health Survey (GDHS), implemented by the Ghana Statistical Service (GSS) with the Ghana Health Service (GHS) and ICF International. The GDHS uses a stratified two-stage cluster sampling design. In the first stage, enumeration areas (primary sampling units, PSUs) were selected from the 2021 Population and Housing Census frame with probability proportional to size; in the second stage, households were systematically selected within each PSU. Trained measurers obtained anthropometry from all children born within five years preceding the survey using standardized protocols. Further detail is available at: www.dhsprogram.com.

### Study population, eligibility, and sample flow

The target population comprised children under five years born to mothers aged 15–24 years (adolescent mothers 15–19 and young adult mothers 20–24). Women aged 25 years and older were excluded. Restriction rather than covariate adjustment was chosen because the study hypothesis concerns the WASH–nutrition relationship within this stratum and does not assume a common effect across maternal age groups. Where two or more eligible children were present, only the index (most recent) child was retained. Children with missing anthropometry (z-scores coded 9,998) or biologically implausible z-scores (HAZ < −6 or > + 6; WHZ < −5 or > + 5; WAZ < −6 or > + 5 SD) were excluded following WHO criteria.

Of 739 initially eligible child–mother pairs, 84 were excluded for missing anthropometry and 35 for implausible z-scores, yielding a final analytic sample of 620 pairs distributed across all 16 regions. Because approximately 16% of eligible pairs were excluded, we compared included and excluded pairs on key covariates (region, wealth, residence and WASH access) to assess the potential for selection bias; results are reported in the Results (Attrition) and Supplementary Table S2. All analyses use this single analytic sample.

### Outcome variables

Three binary outcomes were defined per the WHO Child Growth Standards (33, 34): stunting (HAZ < −2 SD), wasting (WHZ < −2 SD) and underweight (WAZ < −2 SD). DHS z-scores are multiplied by 100; each outcome was coded 1 if the relevant z-score was < −200 and 0 otherwise.

### Exposure and covariate variables

WASH variables followed the WHO/UNICEF Joint Monitoring Programme (JMP) 2021 service ladders (35). Improved water and improved sanitation were coded 1 for JMP basic or safely managed thresholds and 0 otherwise; clean cooking fuel followed the WHO SDG 7.1.2 framework. Maternal characteristics included age, education, marital status, current breastfeeding and media exposure. Household characteristics included residence and the DHS wealth index (asset-based principal-components quintiles); child characteristics were sex and age; the geographic variable was region. For the Concentration Index analyses, the underlying continuous DHS wealth index factor score and completed years of maternal schooling were used rather than their categorical counterparts.

### Survey weighting and complex survey design

All analyses incorporated the complex survey design. The weight v005 was divided by 1,000,000 and applied as a probability weight; v021 was the cluster (PSU) variable and v023 the stratum variable, with singleton strata centered rather than dropped. In the multilevel models, stratification was accommodated by including region as a fixed-effect covariate, consistent with practice for complex-survey multilevel analysis where strata-aware mixed estimation is unavailable in standard melogit syntax (36, 37); cluster-robust standard errors (vce cluster v001) accounted for residual intra-PSU correlation beyond the random intercept. The number of PSU clusters contributing to each fully adjusted model was 206 (stunting), 196 (wasting) and 204 (underweight).

### Statistical analysis

Analyses used Stata MP 17.0 and R 4.5.2. A single estimation procedure was designated authoritative: all prevalence estimates derive from survey-weighted estimation on the analytic sample, so the Abstract and Table 1 report identical figures. All survey-weighted prevalences are accompanied by design-based 95% confidence intervals obtained by Taylor linearisation, reported for the overall sample and for every regional and subgroup estimate.

**Table 1:** Participant characteristics of the analytic sample (*n* = 620).

Characteristic	Weighted count (*n*)	Weighted %
Child nutritional status		
Stunting (HAZ < −2 SD): Yes	116	18.77
Wasting (WHZ < −2 SD): Yes	54	8.65
Underweight (WAZ < −2 SD): Yes	96	15.56
Water, sanitation and hygiene		
Improved water source	513	82.81
Improved sanitation	316	50.94
Clean cooking fuel	62	10.00
Maternal characteristics		
Age 15–19/20–24 years	122/498	19.62/80.38
Education: none/primary/secondary/higher	78/126/408/8	12.63/20.27/65.76/1.34
Marital: never/married/widowed-divorced-separated	199/389/32	32.06/62.78/5.16
Media exposure: yes	503	81.14
Currently breastfeeding: yes	388	62.55
Household characteristics		
Wealth: poorest/poorer/middle/richer/richest	174/160/149/95/42	28.06/25.75/24.00/15.39/6.80
Residence: urban/rural	248/373	39.92/60.08
Child characteristics		
Sex: male/female	315/305	50.84/49.16
Age: <6/6–11/12–35/36–47/48–59 mo	120/125/299/55/21	19.29/20.22/48.16/8.90/3.43

Percentages may not sum to 100% due to rounding. Regional composition is shown with design-based confidence intervals in Table 2. The higher-education stratum comprises only about 5 children (unweighted) and 1.34% of the weighted sample; estimates for this group are correspondingly imprecise.

#### Descriptive analysis

Survey-weighted frequencies and proportions were computed for all characteristics. Design-based chi-squared tests with the Rao–Scott second-order correction assessed bivariate associations. Because Table 2 involves multiple bivariate tests across 13 variables and three outcomes, we interpret individual *p*-values cautiously and additionally report Benjamini-Hochberg false-discovery-rate–adjusted *p*-values in Supplementary Table S3. No bivariate association survived adjustment for multiple comparisons.

**Table 2:** Regional weighted prevalence with design-based 95% confidence intervals and unweighted denominators.

Region	*n*	Stunting % (95% CI)	Wasting % (95% CI)	Underweight % (95% CI)
Western	36	8.2 (3.0–21.4)	7.4 (1.6–24.5)	6.0 (1.6–22.2)
Central	40	16.9 (8.4–31.3)	6.6 (2.7–17.7)	7.4 (2.4–17.3)
Greater Accra	20*	14.0 (3.6–39.8)	10.4 (2.2–35.1)	6.3 (0.8–34.5)
Volta	20*	32.0 (14.4–56.8)	16.8 (5.4–42.9)	39.4 (18.8–62.9)
Eastern	38	13.2 (5.5–28.6)	8.2 (2.4–24.2)	22.7 (11.9–39.4)
Ashanti	48	19.3 (10.9–30.7)	14.3 (6.7–26.5)	19.3 (9.9–32.5)
Western North	25*	17.5 (7.2–37.5)	0.0 (—)	9.7 (2.3–32.2)
Ahafo	48	25.1 (16.1–36.8)	7.6 (2.9–18.9)	16.1 (9.6–26.4)
Bono	21*	10.5 (2.9–31.8)	6.3 (0.8–32.9)	5.9 (0.8–29.2)
Bono East	43	11.0 (5.0–23.7)	0.6 (0.2–8.3)	10.3 (4.2–21.5)
Oti	58	24.3 (15.5–34.6)	12.5 (6.9–22.6)	21.6 (13.8–33.6)
Northern	61	27.6 (15.7–43.7)	8.5 (3.9–14.8)	26.4 (14.7–41.5)
Savannah	60	21.1 (10.4–37.9)	3.3 (0.4–22.6)	6.9 (2.0–21.2)
North East	81	25.4 (14.4–40.3)	13.8 (7.2–25.2)	28.1 (16.6–42.8)
Upper East	52	23.1 (12.2–39.8)	2.3 (0.2–13.3)	8.0 (1.9–29.2)
Upper West	48	21.4 (11.7–35.7)	5.9 (1.9–16.9)	15.9 (6.9–31.7)

Weighted row percentages with Taylor-linearised design-based 95% confidence intervals; *n* is the unweighted regional denominator in the analytic sample. * Unweighted *n* < 30: estimate statistically unstable and imprecise. Wasting was not observed in Western North (0%), so no interval is defined. Confidence intervals are wide for small regions, and regional rankings based on them should be treated as tentative.

#### Multilevel logistic regression

Survey-weighted two-level models with random intercepts at PSU level were fitted for each outcome. For each outcome a null (intercept-only) model partitioned variance into within- and between-PSU components; the intra-cluster correlation coefficient (ICC) was σ^2^u/(σ^2^u + π^2^/3). Throughout, σ^2^u in the ICC and MOR formulae refers exclusively to the null-model PSU-level variance σ^2^u(null), and the proportional change in variance is PCV = [σ^2^u(null) − σ^2^u(full)]/σ^2^u(null) (38). A fully adjusted model then incorporated all WASH exposures and covariates as fixed effects; adjusted odds ratios (aOR) with 95% CIs were obtained by exponentiation. Model fit used AIC, BIC and McFadden’s pseudo-R^2^ (0.10–0.20 acceptable, ≥ 0.20 good, thresholds lower than for linear R^2^ because of the constrained variance of binary outcomes (39)). Multicollinearity was assessed with Generalized Variance Inflation Factors; GVIF^(1/(2 × df)) values ranged from 1.04 (child sex) to 3.87 (region), with per-predictor values in Supplementary Table S1. The median odds ratio (MOR) was exp.(√(2σ^2^u) × Φ^−1^(0.75)). Discrimination used the area under the ROC curve (AUC).

#### Spatial analysis

Regional weighted prevalences were merged with a validated Ghana regional shapefile. A queen-contiguity spatial weights matrix (row-standardized) accommodated regions separated by Lake Volta. Global Moran’s I tested spatial randomness. Local Indicators of Spatial Association (LISA) and Getis-Ord G* were computed but are reported as exploratory, hypothesis-generating analyses: with only 16 areal units, both global and local statistics have limited power and local *p*-values may be unstable at the tails, so the possibility of Type I error is non-trivial. To gauge stability, LISA classifications were re-assessed with a leave-one-region-out jackknife (Supplementary Table S4). The Oti underweight hotspot persisted in most replicates, whereas smaller-sample classifications were less stable. As a robustness check for Objectives 2 and 3, we repeated the analysis excluding regions with unweighted *n* < 20 and pooling newly created regions into their historical parents.

#### Inequality measures

Population Attributable Fraction and Risk. For each binary WASH exposure, PAF used Levin’s formula PAF = Pe(RR − 1)/[1 + Pe(RR − 1)], with an alternative formulation PAF = (Pall − Punexposed)/Pall; PAR = Pall − Punexposed. Negative PAF indicates a protective exposure. PAF and PAR are reported for all exposure × outcome combinations (Table 3). PAF estimates are population-level attributable-burden quantities under a cross-sectional design and are not causal effect sizes.

**Table 3:** Completed PAF and PAR for all binary exposure × outcome combinations.

Outcome	Exposure	P_exp %	P_unexp %	RR	PAF (Levin) %	PAR (pp)
Stunting	Improved water	17.3	25.6	0.68	−36.4	−6.8
Stunting	Improved sanitation	16.5	21.1	0.78	−12.4	−2.3
Stunting	Clean cooking fuel	17.8	18.9	0.94	−0.6	−0.1
Stunting	Breastfeeding ᵃ	17.6	20.7	0.85	−10.6	−2.0
Underweight	Improved water	14.4	21.4	0.67	−37.5	−5.8
Underweight	Improved sanitation	11.7	19.6	0.59	−26.1	−4.1
Underweight	Clean cooking fuel	12.6	15.9	0.79	−2.1	−0.3
Underweight	Breastfeeding ᵃ	15.7	15.3	1.03	+1.6	+0.2
Wasting	Improved water	8.5	9.3	0.92	−7.3	−0.6
Wasting	Improved sanitation	7.5	9.8	0.76	−13.7	−1.2
Wasting	Clean cooking fuel	10.2	8.5	1.20	+2.0	+0.2
Wasting	Breastfeeding ᵃ	11.0	4.7	2.33	+45.5	+3.9

Weighted estimates on the analytic sample. Negative PAF/PAR indicate a protective exposure. 𝐻reastfeeding associations reflect reverse causality and are not causal; positive PAF values for breastfeeding should not be interpreted as harm.

#### Difference Index (DI) and Relative Index (RI)

Survey-weighted prevalence was estimated by subgroup. To avoid an unstable reference, the reference for each outcome was the subgroup with the lowest observed prevalence among subgroups with an unweighted denominator of at least 30; the higher-education stratum (unweighted *n* ≈ 5) was therefore not used as a reference and its comparison is retained only as a sensitivity analysis (Supplementary Table S6). DI = Pgroup − Preference (percentage points); RI = Pgroup/Preference.

Theil T Index: T = Σ sg(μg/μ)ln(μg/μ), computed for wealth quintile, education, residence and region. Because the Theil index is defined over discrete subgroups and requires no rank ordering, it is the appropriate measure for the nominal groupings (region and residence).

For Wagstaff Concentration Index CI = (2/μ) × Cov(y, r), with the Wagstaff-normalised index CIw = CI/(1 − μ) for binary outcomes (40); 95% CIs used CI ± 1.96 × SE (27). The Concentration Index requires a continuous (or meaningfully ordinal) ranking variable because the fractional rank must be defined along a single socioeconomic gradient (41, 42). Accordingly, it was computed on two continuous axes only: the DHS wealth index factor score and completed years of maternal schooling. Place of residence (binary) and region (16 unordered categories) carry no natural socioeconomic rank and are instead assessed with the Theil T index. Concentration curves plotted the cumulative outcome share against the cumulative population share ranked by each axis. Wagstaff’s decomposition disaggregated the CI into determinant contributions Ck = ηk × CIxk; the restriction to continuous axes applies to the ranking axis, not to the determinants, so region and residence remain valid determinants within the decomposition.

### Ethical considerations

The 2022 GDHS received ethical clearance from the Ghana Health Service Ethics Review Committee and the ICF Institutional Review Board. Consent followed an age-based procedure consistent with GDHS protocols: written informed consent was obtained from participants aged 18 years and older, and for participants below 18 years, parental (or guardian) consent together with the adolescent’s assent was obtained. The manuscript and the submission-system ethics statement have been harmonized to reflect this age-based consent and assent procedure. This analysis used fully anonymized, publicly available secondary data accessed via https://dhsprogram.com following registration; no additional ethics approval was required. The study followed the Declaration of Helsinki (2013) and STROBE reporting guidelines; the completed STROBE checklist is provided as Supplementary material.

#### Relationship to companion work

Two companion analyses (26, 28) share authorship and the GDHS data source. The present analytic sample is restricted to index children of mothers aged 15–24 years (*n* = 620) and addresses stunting, wasting and underweight in this subpopulation; it is distinct in sample, outcomes and research questions from reference (26) (sexual autonomy among married young women) and reference (28) (household water and sanitation and diarrhoeal/acute respiratory infection among all under-five children). There is no duplication of the spatial, inequality or regression results across these papers.

## Results

### Descriptive analysis

Table 1 presents the weighted characteristics of the analytic sample of 620 children born to mothers aged 15–24 years. Stunting prevalence was 18.77% (95% CI 15.5–22.6), wasting 8.65% (6.2–11.9), and underweight 15.56% (12.4–19.4). For WASH, 82.81% of households had improved water, 50.94% improved sanitation, and 10.00% clean cooking fuel. Most mothers were aged 20–24 years (80.38%), had secondary education (65.76%), were married or cohabiting (62.78%), and had media exposure (81.14%). Household wealth skewed poorer (28.06% poorest; 6.80% richest); 60.08% of children lived in rural areas; 50.84% were male; and most were aged 12–35 months (48.16%).

### Prevalence of stunting, wasting, and underweight by region

**Figure 1:**
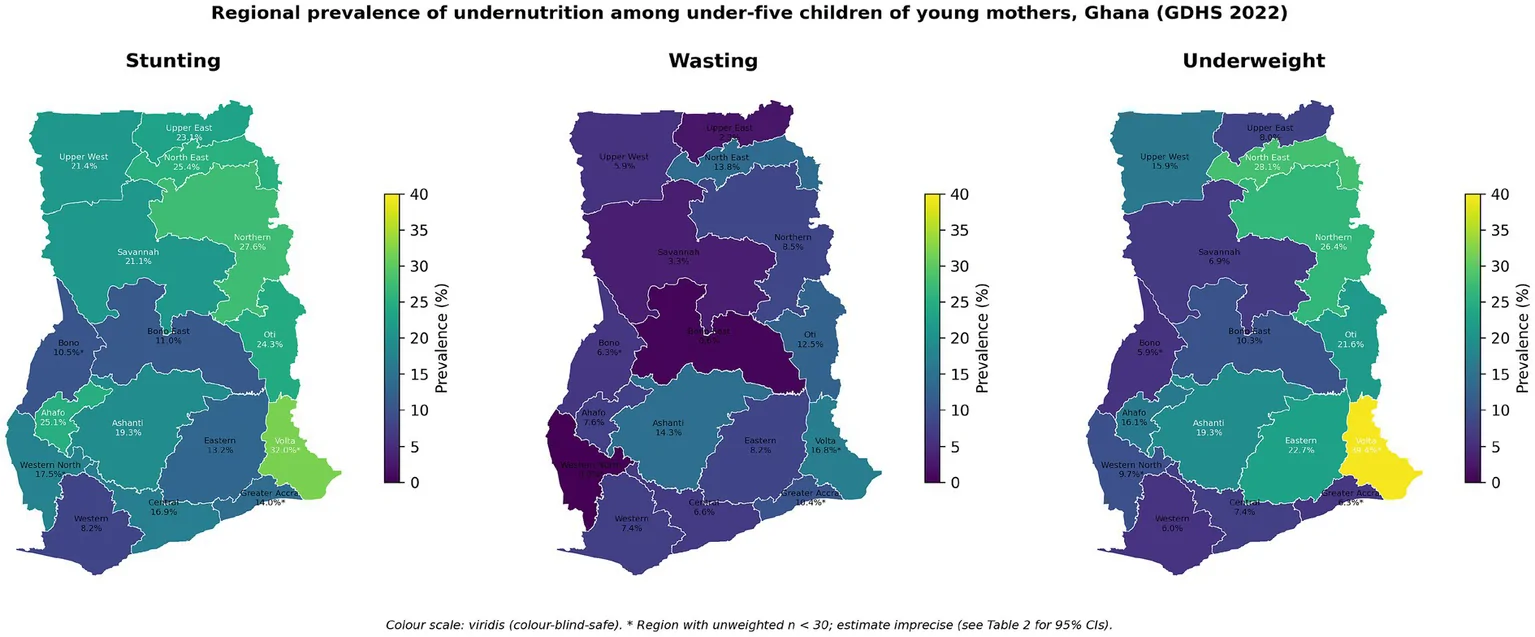
Regional prevalence of stunting, wasting, and underweight among under-five children of young mothers (GDHS 2022). Color-blind-safe viridis scale; Regions with unweighted *n* < 30 are marked with an asterisk and should be interpreted as imprecise (see Table 2).

Figure 1 maps the survey-weighted regional prevalence. Point estimates are accompanied by design-based 95% confidence intervals and unweighted denominators in Table 2. Several regions have small subsamples, and their estimates are correspondingly imprecise. Stunting ranged from 8.2% in Western to 32.0% in Volta; underweight from 5.9% in Bono to 39.4% in Volta; and wasting from effectively 0% in Western North to 16.8% in Volta. Volta recorded the highest observed weighted prevalence for all three outcomes, but this estimate was based on a small subsample (unweighted *n* = 20) and is imprecisely estimated (stunting 95% CI 14.4–56.8; underweight 18.8–62.9); it should therefore be read as the highest observed rather than as an established ranking. A broad north–south gradient was apparent for stunting.

### Attrition: comparison of included and excluded pairs

Of 739 eligible pairs, 119 (16.1%) were excluded for missing or implausible anthropometry (84 and 35 respectively). Included and excluded pairs did not differ significantly on wealth quintile (χ^2^ *p* = 0.91) or urban–rural residence (*p* = 0.92). Excluded pairs were somewhat more likely to lack improved water (*p* = 0.033) and showed borderline differences by region (*p* = 0.079) and improved sanitation (*p* = 0.083). This pattern indicates that missingness was not entirely at random with respect to WASH access. We therefore interpret WASH-related distributions with corresponding caution and note that any resulting bias would most plausibly attenuate rather than inflate the observed WASH–nutrition gradients. Full comparisons are in Supplementary Table S2.

### Local spatial analysis: LISA and Getis-Ord G* (exploratory)

**Figure 2:**
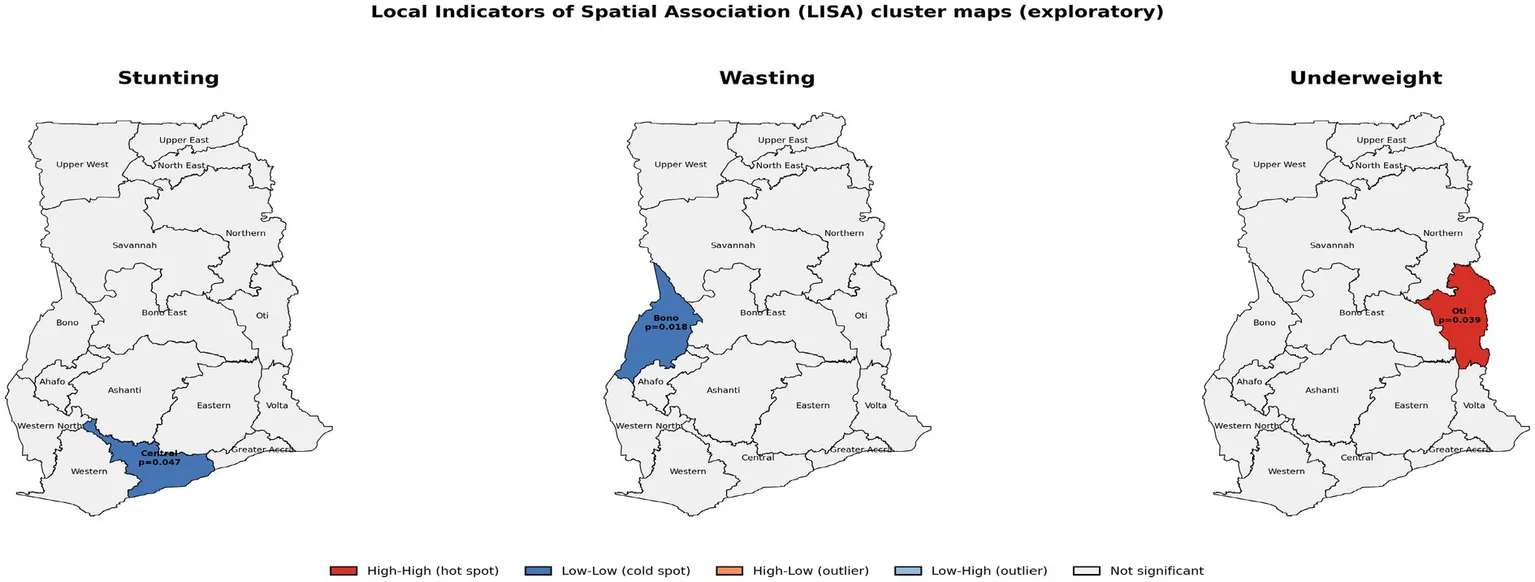
LISA cluster maps for stunting, wasting and underweight (exploratory). Color-blind-safe categorical palette; only regions significant at *p* < 0.05 are shaded.

The local spatial statistics below are reported as exploratory, hypothesis-generating findings. With only 16 areal units, local *p*-values can be unstable at the tails and Type I error is a real possibility. Thus, these results should not be read as confirmatory. In LISA analysis (Figure 2), Central emerged as a low–low cold spot for stunting (*p* = 0.047), Bono as a low–low cold spot for wasting (*p* = 0.018), and Oti as a high–high hot spot for underweight (*p* = 0.039). Ashanti yielded an undefined local statistic for stunting and underweight, consistent with computational instability at near-average prevalence. No spatial outliers were significant. A leave-one-region-out jackknife (Supplementary Table S4) indicated that the Oti underweight hotspot remained significant in most replicates, whereas cold-spot classifications in smaller-sample regions were less stable.

**Figure 3:**
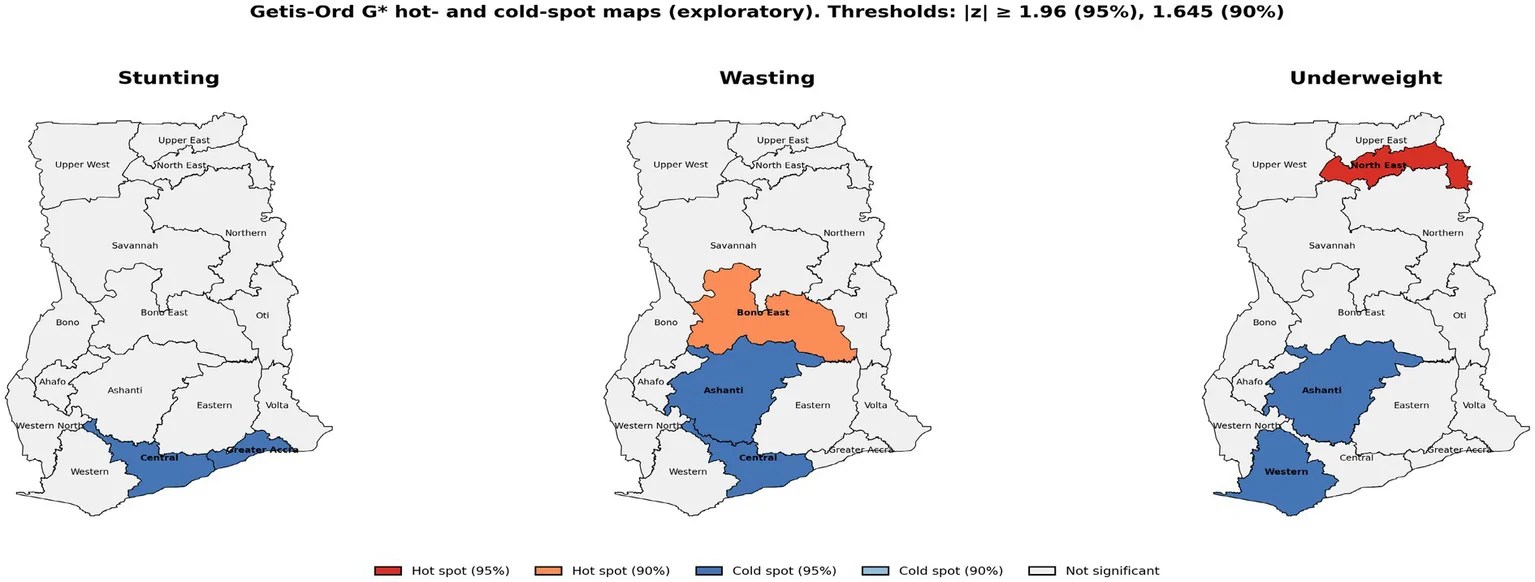
Getis-Ord G* hot- and cold-spot maps (exploratory). Thresholds |*z*| ≥ 1.96 (95%) and 1.645 (90%).

The Getis-Ord G* statistic (Figure 3) identified a south-central cold-spot corridor (Greater Accra and Central) for stunting; Ashanti and Central cold spots for wasting with a marginal middle-belt hot signal (90%); and, for underweight, North East as a hot spot (95%) with Ashanti and Western as cold spots.

#### Reconciling LISA and Getis-Ord

The two statistics answer different questions and are expected to diverge. LISA (local Moran’s I) flags a region whose value co-varies with its neighbors’ values relative to the global mean, so it highlighted Oti, whose above-average underweight prevalence is shared with contiguous neighbors. Getis-Ord G* instead sums a region’s value together with its neighbors’ values and flags where that local sum is extreme; North East, embedded among other high-underweight northern regions, produced the most extreme local sum. The divergence therefore reflects the distinction between local spatial association (LISA) and local intensity (G*) rather than a contradiction. Because both are exploratory here, we do not privilege either single-region signal.

### Global spatial autocorrelation

**Figure 4:**
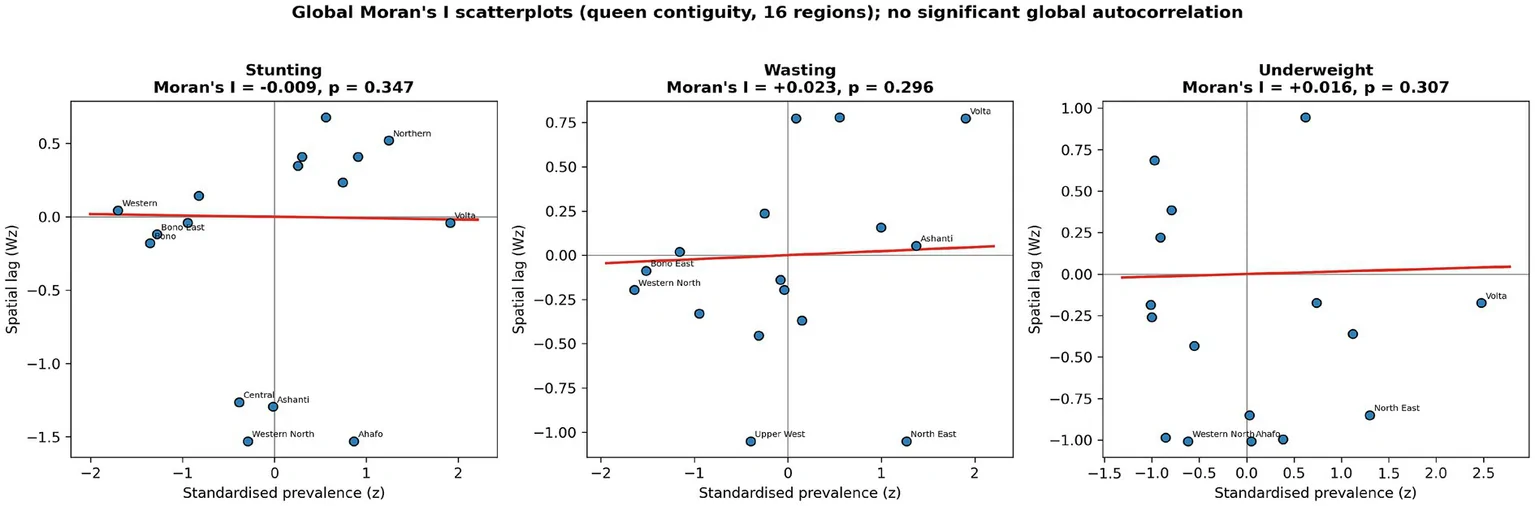
Global Moran’s I scatterplots (queen contiguity, 16 regions). Near-flat slopes and non-significant *p*-values indicate the absence of global spatial autocorrelation.

Global Moran’s I indicated no statistically significant global spatial autocorrelation for any outcome (Figure 4): stunting I = −0.009 (*p* = 0.347), wasting I = 0.023 (*p* = 0.296), and underweight I = 0.016 (*p* = 0.307). The correct minimum bound across the three outcomes is therefore *p* ≥ 0.296. The regional distribution of malnutrition in this subpopulation is thus not systematically clustered at the national scale but is organized into discrete local pockets. With only 16 spatial units, the power of the global test is limited, and this null result should not be read as evidence that no spatial pattern exists.

### Bivariate analysis of factors associated with nutritional outcomes

Table 2 (bivariate) shows weighted associations. Improved sanitation was associated with lower underweight prevalence (11.6% vs. 20.1%, *p* = 0.044); improved water approached significance for stunting (*p* = 0.057) and underweight (*p* = 0.080). Regional differences reached significance only for underweight (*p* = 0.031), residence for underweight (*p* = 0.045), and wealth for underweight (*p* = 0.001). Wasting varied by current breastfeeding (11.1% vs. 4.6%, *p* = 0.046).

Table 2 comprises multiple bivariate tests across 13 variables and three outcomes. After Benjamini-Hochberg control of the false discovery rate, no bivariate association remained significant (Supplementary Table S3). The nominal associations above should therefore be regarded as hypothesis-generating rather than confirmatory. The unadjusted bivariate table is provided in full in Supplementary Table S3.

### Measures of association

Household water, sanitation and cooking fuel were not significantly associated with any outcome in the fully adjusted models (Table 4); adjusted odds ratios for improved water ranged from 0.67 (stunting) to 1.61 (wasting), all confidence intervals crossing the null. Children of mothers aged 20–24 had higher odds of wasting than those of adolescent mothers (aOR 3.56; 95% CI 1.14–11.08). Children from the second-poorest households had higher odds of underweight than the poorest (aOR 2.55; 1.19–5.48). Two associations are flagged as likely artefacts and are not interpreted causally (see footnotes to Table 4): the significantly lower odds of stunting and underweight among children of widowed, divorced or separated mothers (aOR ≈ 0.14–0.15), and the higher odds of underweight among currently breastfeeding children (aOR 3.23). The higher-education stratum yields extremely wide, uninformative estimates (e.g., wasting aOR 7.85; 95% CI 0.32–190.27) because it contains only about 5 children, and no inference is drawn from it.

**Table 4:** Adjusted odds ratios from survey-weighted multilevel logistic regression.

Predictor	Stunting aOR (95% CI)	Underweight aOR (95% CI)	Wasting aOR (95% CI)
Improved water (vs unimproved)	0.67 (0.32–1.40)	1.13 (0.49–2.63)	1.61 (0.42–6.17)
Improved sanitation (vs unimproved)	1.18 (0.56–2.50)	0.63 (0.27–1.48)	0.46 (0.13–1.60)
Clean cooking fuel (vs unclean)	1.99 (0.52–7.67)	3.06 (0.81–11.51)	2.36 (0.34–16.59)
Maternal age 20–24 (vs 15–19)	0.90 (0.48–1.71)	1.33 (0.64–2.73)	3.56 (1.14–11.08)
Rural (vs urban)	0.78 (0.38–1.62)	1.48 (0.70–3.13)	1.30 (0.46–3.64)
Wealth: poorer (vs poorest)	1.41 (0.69–2.86)	2.55 (1.19–5.48)	1.51 (0.50–4.58)
Wealth: middle	0.63 (0.22–1.79)	0.77 (0.26–2.27)	0.98 (0.20–4.78)
Wealth: richer	0.85 (0.22–3.31)	0.67 (0.17–2.70)	0.19 (0.02–1.78)
Wealth: richest	0.79 (0.13–4.98)	0.08 (0.01–1.19)	0.22 (0.01–5.38)
Female (vs male)	0.64 (0.33–1.22)	0.72 (0.38–1.36)	0.51 (0.23–1.13)
Education: primary (vs none)	0.76 (0.33–1.74)	0.58 (0.22–1.55)	0.69 (0.19–2.52)
Education: secondary	0.62 (0.28–1.39)	0.53 (0.23–1.23)	0.69 (0.20–2.36)
Education: higher ᵃ	0.60 (0.04–10.35)	3.50 (0.22–55.76)	7.85 (0.32–190.27)
Married/cohabiting (vs never married)	0.43 (0.20–0.93)	0.40 (0.14–1.12)	0.69 (0.18–2.63)
Widowed/divorced/separated ᵇ	0.15 (0.04–0.62)	0.14 (0.03–0.80)	0.22 (0.02–2.17)
Media exposure (vs none)	1.19 (0.55–2.57)	1.30 (0.57–2.94)	0.81 (0.33–2.02)
Child age 6–11 mo (vs < 6)	0.55 (0.23–1.35)	1.52 (0.52–4.43)	4.91 (1.27–19.07)
Child age 12–35 mo	1.64 (0.76–3.51)	3.88 (1.56–9.64)	2.19 (0.58–8.31)
Child age 36–47 mo	1.44 (0.42–4.98)	8.60 (2.04–36.21)	0.82 (0.08–8.44)
Child age 48–59 mo	1.34 (0.18–10.09)	7.95 (0.74–85.59)	0.99 (0.05–20.78)
Currently breastfeeding ᵇ	1.10 (0.58–2.10)	3.23 (1.35–7.72)	3.66 (0.86–15.59)

aOR from survey-weighted two-level mixed-effects logistic regression with a PSU-level random intercept; models adjust for all variables shown plus region. PSU clusters: stunting 206; underweight 204; wasting 196. ᵃThe higher-education stratum contains only ~5 children; its estimates are uninformative (very wide intervals) and are not interpreted. 𝑿lagged as likely reverse causality/selection: acutely ill children are commonly kept on breastfeeding, and marital-status effects most plausibly reflect selection. These associations are not interpreted causally (see Discussion).

The marginal bivariate association between improved water and underweight (21.3% vs. 14.4%) attenuated to the null in the adjusted model (aOR ≈ 1.13). Sequential adjustment indicated that this attenuation was driven principally by wealth quintile and region, the covariates most strongly correlated with both water access and underweight. This is consistent with the concentration-index decomposition (Table 5), in which improved water and region are the leading contributors to the wealth-axis concentration of stunting.

**Table 5:** Decomposition of the wealth-axis Concentration Index for stunting (CI = −0.043).

Determinant	Elasticity	Contribution to CI	% contribution
Region	+0.367	−0.064	148.3
Improved water	−0.265	−0.047	110.3
Maternal education	−0.244	−0.030	69.0
Improved sanitation	−0.048	−0.019	44.8
Child age	+0.383	−0.003	6.9
Child sex (female)	−0.403	−0.002	3.4
Maternal age	+0.131	+0.002	−3.4
Marital status	−0.331	+0.016	−37.9
Media exposure	+0.183	+0.027	−62.1
Clean cooking fuel	+0.027	+0.028	−65.5
Residence (rural)	−0.339	+0.049	−113.8
Total		−0.043	100.0

### Measures of variation

Table 6 presents the variance components. All ICC, MOR and PCV statistics derive from the null-model PSU-level variance. Between-cluster variance was substantial for all outcomes (null-model σ^2^u 1.61–2.28; ICC 30–41%; MOR 3.14–4.18), yet covariates explained only 1.4–24.8% of that variance, indicating that a large share of contextual heterogeneity is unmeasured. McFadden pseudo-R^2^ values were 0.146 (stunting), 0.203 (underweight) and 0.198 (wasting), spanning acceptable to good fit.

**Table 6:** Measures of variation and model fit (null and adjusted models combined).

Measure	Stunting	Underweight	Wasting
Null-model deviance (−2LL₀)	644.86	475.81	383.32
Adjusted-model deviance (−2LLf)	550.45	447.38	307.21
Likelihood-ratio χ^2^ (−2LLR)	94.41	28.43	76.11
AIC/BIC (adjusted)	626.5/799.7	523.4/696.6	381.2/548.4
PSU variance, null σ^2^u(null)	1.613	1.933	2.278
PSU variance, adjusted σ^2^u(full)	1.433	1.461	2.245
ICC (from null model)	30.3%	30.8%	40.6%
MOR (from null model)	3.14	3.17	4.18
PCV	11.2%	24.8%	1.4%
McFadden pseudo-R^2^	0.146	0.203	0.198

ICC and MOR computed from σ^2^u(null); PCV = [σ^2^u(null) − σ^2^u(full)]/σ^2^u(null). Both null- and adjusted-model variances are shown.

### Predictive performance of the models

**Figure 5:**
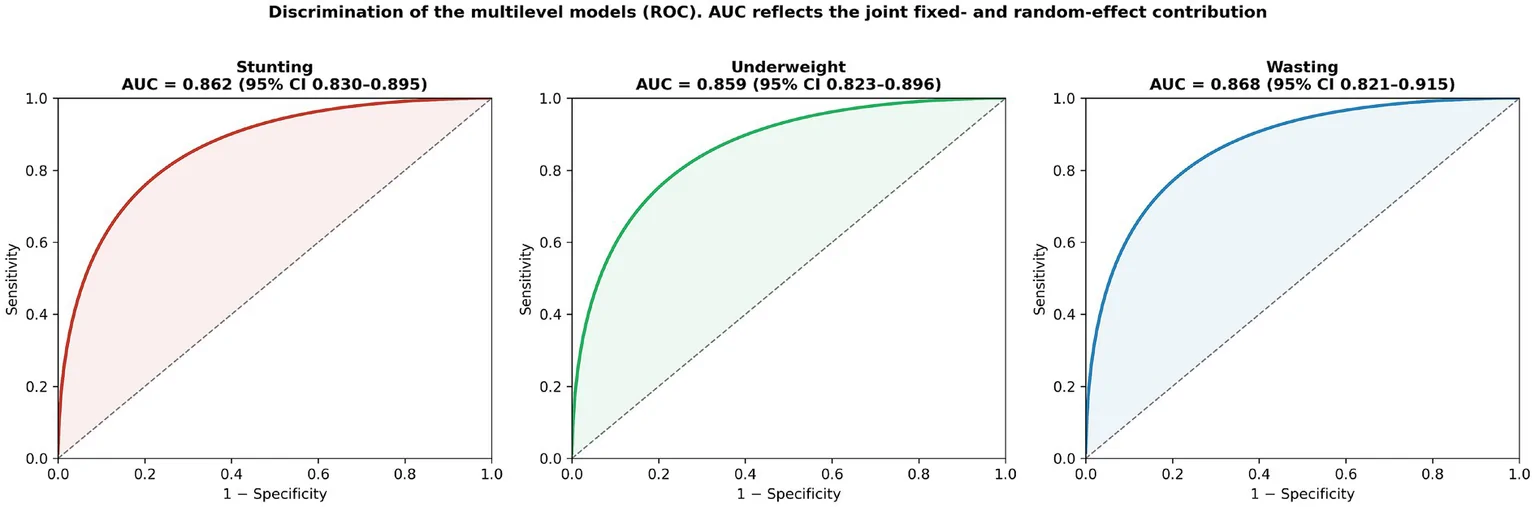
Discrimination (ROC) of the three multilevel models. AUC reflects the combined fixed- and random-effect structure.

The AUC was 0.862 (95% CI 0.830–0.895) for stunting, 0.859 (0.823–0.896) for underweight, and 0.868 (0.821–0.915) for wasting (Figure 5). These high values reflect the joint contribution of the measured fixed effects and the unmeasured PSU-level heterogeneity captured by the random intercept, and do not by themselves validate the sufficiency of the measured covariate set. This caution is reinforced by the PCV statistics, which show that covariates explain only 1.4–24.8% of between-PSU variance.

### Difference index and relative index

Table 7 has been computed using a stable reference category (the lowest-prevalence subgroup with an unweighted denominator of at least 30). Inequalities remained pronounced. For underweight, the poorer quintile (26.8%) exceeded the middle quintile (8.2%), a Relative Index of 3.28; the education gradient (no education 25.1% vs. secondary-or-higher 13.6%) gave RI 1.85; and children without improved sanitation had 1.68 times the underweight prevalence of those with it. For stunting, no education carried 1.58 times the prevalence of secondary-or-higher schooling and unimproved water 1.48 times the prevalence of improved water. The higher-education comparison is retained only as a sensitivity analysis (Supplementary Table S6).

**Table 7:** Difference and relative indices using a stable reference subgroup (unweighted *n* ≥ 30).

Outcome	Highest-burden group	*n*	Reference (stable)	DI (pp)	RI
Stunting	Wealth: poorer (23.4%)	*n* = 193	Middle (12.9%)	10.5	1.81
Stunting	No education (27.3%)	*n* = 132	Secondary+ (17.3%)	10.0	1.58
Stunting	Unimproved water (25.6%)	*n* = 165	Improved (17.3%)	8.2	1.48
Underweight	Wealth: poorer (26.8%)	*n* = 193	Middle (8.2%)	18.6	3.28
Underweight	No education (25.1%)	*n* = 132	Secondary+ (13.6%)	11.5	1.85
Underweight	Unimproved sanitation (19.6%)	*n* = 434	Improved (11.7%)	8.0	1.68
Wasting	Wealth: poorer (10.8%)	*n* = 193	Richer (5.8%)	5.0	1.88
Wasting	Unimproved sanitation (9.8%)	*n* = 434	Improved (7.5%)	2.3	1.31

Reference = lowest-prevalence subgroup with unweighted *n* ≥ 30, ensuring a stable denominator. DI, percentage-point difference; RI, ratio of prevalences; pp = percentage points.

### Population attributable fraction and population attributable risk

Table 3 reports PAF and PAR for all exposure × outcome combinations. Improved water carried the largest protective population-level association for stunting and underweight (Levin PAF − 36 to −41%), reflecting both a favorable prevalence ratio and high coverage. Improved sanitation was protective across all three outcomes (PAF − 12 to −27%), with impact limited by its lower coverage (50.9%). Clean fuel effects were negligible given very low coverage (10.0%). The positive PAF for current breastfeeding and wasting (Levin PAF ≈ +46%) is a recognized artefact of reverse causality (acutely wasted children are commonly kept on or returned to breastfeeding) and is not evidence of harm. All PAF/PAR values are population-level attributable-burden estimates under a cross-sectional design and are not causal effect sizes.

### Theil T index

Regional variation dominated inequality in wasting (T = 0.151) and contributed substantially to underweight (0.143) and stunting (0.050). Wealth contributed most to underweight (0.117); residence contributed little to any outcome. Because the Theil index requires no rank ordering, it is the appropriate instrument for the nominal groupings (region, residence).

### Wagstaff Concentration Index and concentration curves

**Figure 6:**
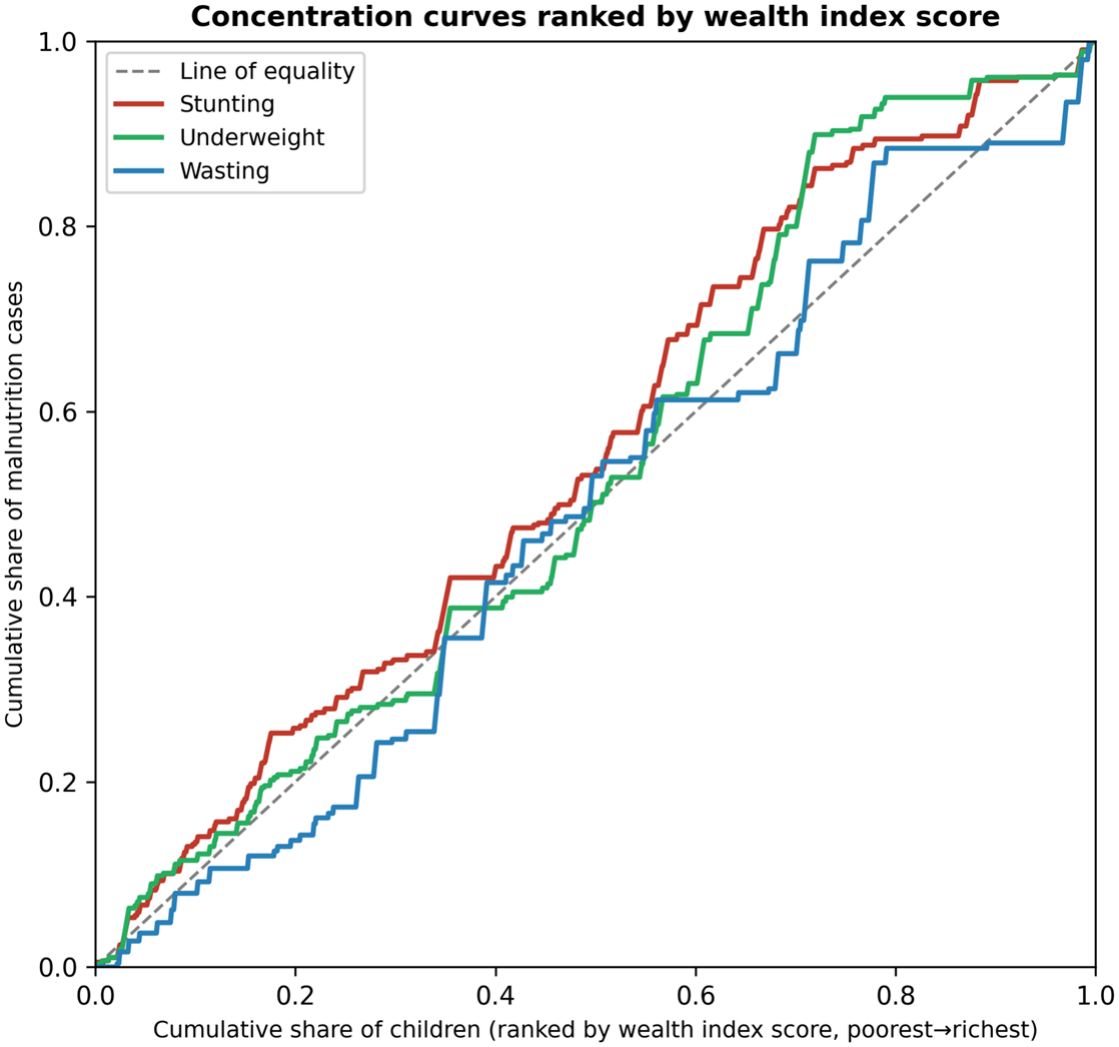
Concentration curves ranked by household wealth index score. Curves above the diagonal indicate concentration of malnutrition among poorer households.

**Figure 7:**
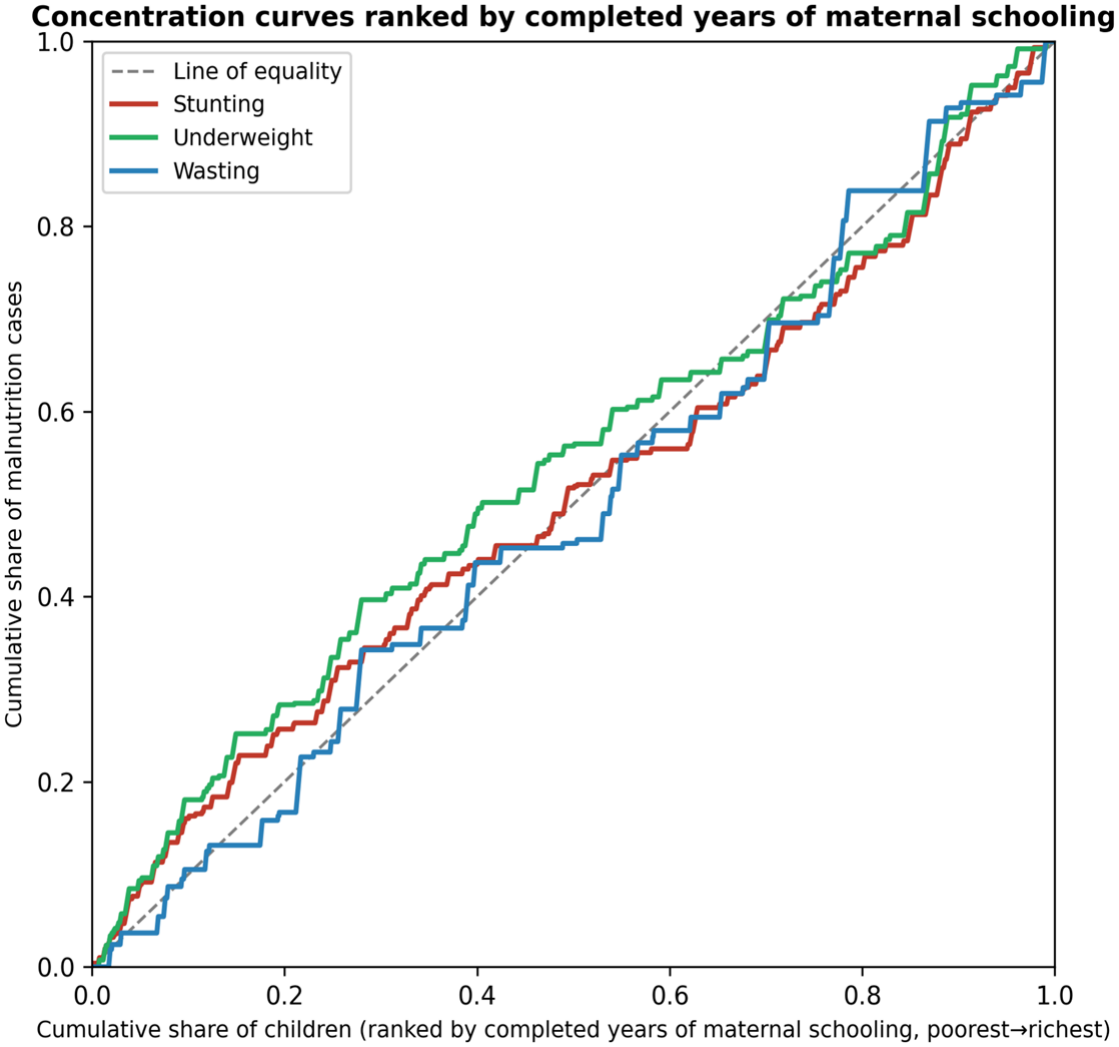
Concentration curves ranked by completed years of maternal schooling. Curves above the diagonal indicate concentration of malnutrition among less-educated households.

On the two continuous ranking axes (wealth index factor score and completed years of maternal schooling), concentration indices were negative for all outcomes, confirming pro-poor and pro-less-educated concentration (Table 8). Underweight was the only outcome for which the 95% CI excluded zero on both axes. Residence and region were excluded as ranking axes because they carry no natural socioeconomic order. They are assessed via the Theil T index (Table 9). Figures 6, 7 confirms this pattern.

**Table 8:** Concentration Index on continuous socioeconomic axes.

Outcome	Ranking axis	CI	95% CI	Wagstaff CIw
Stunting	Wealth index score	−0.043	−0.125 to 0.039	−0.053
Stunting	Years of schooling	−0.036	−0.122 to 0.050	−0.044
Wasting	Wealth index score	−0.110	−0.245 to 0.025	−0.120
Wasting	Years of schooling	−0.046	−0.181 to 0.089	−0.050
Underweight*	Wealth index score	−0.182	−0.272 to −0.092	−0.216
Underweight*	Years of schooling	−0.102	−0.200 to −0.004	−0.121

Negative CI = concentration among the worse-off. CIw = CI/(1 − μ). *95% CI excludes zero (statistically significant concentration). Ranking axes are continuous, per the requirement that the fractional rank be defined along a single socioeconomic gradient.

**Table 9:** Theil T index of between-group inequality.

Grouping variable	Stunting	Underweight	Wasting
Wealth quintile	0.0211	0.1167	0.0169
Maternal education	0.0166	0.0271	0.0220
Residence	0.0000	0.0338	0.0022
Region	0.0502	0.1430	0.1509

Interpretation: < 0.01 minimal; 0.01–0.05 moderate; > 0.05 substantial. Region and residence are nominal groupings for which Theil T (not the Concentration Index) is appropriate.

### Concentration Index decomposition

Table 5 decomposes the wealth-axis Concentration Index for stunting. Improved water and region were the largest contributors to pro-poor concentration, with maternal education third; rural residence, clean fuel and media exposure made offsetting equalizing contributions. Some determinants (e.g., unequal water access) push malnutrition toward poorer children, while others (e.g., the rural distribution of the sample) pull in the opposite direction; because contributions can be positive or negative, an individual contribution can exceed the total Concentration Index in magnitude, and the signed contributions sum to 100% of the index. On the education axis (Table 10), maternal education itself was the dominant contributor, confirming that educational inequality in stunting is structurally embedded in the schooling gradient rather than reducible to wealth or geography.

**Table 10:** Decomposition of the education-axis Concentration Index for stunting (CI = −0.036).

Determinant	Elasticity	Contribution to CI	% contribution
Maternal education*	−0.307	−0.051	141.7
Improved water	−0.265	−0.008	20.8
Region	+0.367	+0.023	−64.6
Media exposure	+0.183	+0.005	−12.5
Other determinants (combined)	—	+0.005	14.6
Total		−0.036	100.0

Contribution = elasticity × CIxk. Positive and negative contributions offset; percentages sum to 100%. Region and residence are valid determinants (each entering through its own Concentration Index relative to the continuous ranking axis) even though neither is a valid ranking axis. * Education is both the ranking axis and a determinant.

## Discussion

This study characterizes the spatial distribution, socioeconomic inequality and WASH-related determinants of stunting, wasting and underweight among under-five children born to young mothers (15–24 years) in Ghana. Three findings emerge. First, the burden of all three forms of malnutrition is substantial and spatially heterogeneous, with the highest observed prevalences in the Volta Belt and northern zones and a broad north–south gradient for stunting. Second, no statistically significant global spatial autocorrelation was detected at regional level, while exploratory local analyses suggested geographic pockets of vulnerability. Third, malnutrition is disproportionately concentrated among the poorest and least-educated households, with WASH deficits, particularly inadequate improved water access, constituting the largest modifiable population-level attributable burden.

The weighted stunting prevalence of 18.8% among children of young mothers is broadly comparable to the 2022 GDHS national estimate of ~17% for all under-five children. This is contextually noteworthy given the documented vulnerabilities of young mothers and may partly reflect the concentrated delivery of preventive services to this age group within Ghana’s CHPS system. Nonetheless, nearly one in five children being stunted remains unacceptably high.

The apparent concentration of burden in Volta and Oti should be read with caution. These regions have small analytic subsamples (unweighted *n* of about 20 and 58, respectively), and their prevalence estimates carry wide confidence intervals (for example, Volta stunting 32.0, 95% CI 14.4–56.8). A single misclassified child in a region of ~20 could shift the estimate by several percentage points. We therefore describe Volta as recording the highest observed weighted prevalence rather than the established highest burden, and treat the Oti underweight hotspot as an exploratory, hypothesis-generating signal that persisted in most but not all jackknife replicates. Volta’s pattern is nonetheless consistent with prior DHS analyses of the Volta Belt as a high-burden zone, driven by dependence on rain-fed agriculture, limited dry-season water, and historically high open defecation (7, 9).

The LISA and Getis-Ord results identify different single regions (Oti versus North East for underweight). As explained above, this reflects the conceptual distinction between local spatial association and local intensity rather than a contradiction; with only 16 units, neither should be over-interpreted. The absence of significant global autocorrelation is consistent with prior regional-level spatial analyses of child nutrition in Ghana, where 16 spatial units limit the power of global Moran’s I (29); comparable studies detecting significant clustering operated at district level with many more units (29, 30).

The PAF estimates are among the most policy-relevant findings. Improved water access yielded a Levin PAF of about −37 to −41% for stunting and underweight, consistent with multi-country evidence that improved water and sanitation are among the most consistently protective factors for child nutrition (17), and with graded WASH–nutrition relationships reported in Benin (21). Plausible mechanisms include reduced fecal-oral transmission, lower diarrhoeal morbidity, improved gut mucosal integrity and reduced systemic inflammation (12–14, 16). However, because the design is cross-sectional, these are attributable-burden estimates, not causal effect sizes, and should be read with the same caution applied to the wide confidence intervals elsewhere.

The positive PAF for current breastfeeding and wasting (≈ + 46%) and the markedly lower odds of stunting/underweight among children of widowed, divorced or separated mothers are biologically implausible as causal effects and most plausibly reflect reverse causality or selection. Acutely wasted or ill children are commonly kept on or returned to breastfeeding; marital-status associations may reflect selective survival, re-partnering, or economic dynamics not captured by status. Both are treated as hypothesis-generating and are not translated into policy guidance.

The socioeconomic inequality findings are internally consistent: negative concentration indices on both continuous axes and across all outcomes confirm pro-poor concentration, statistically significant for underweight. The Theil T evidence that regional variation dominates wasting inequality implies that geographic context may matter more than individual household position for acute malnutrition, while region and wealth jointly drive underweight inequality. The decomposition identifies improved water, region and maternal education as leading contributors to the wealth-axis concentration of stunting, suggesting combined water-infrastructure and girls’-education investment as a coherent equity strategy (43).

In the multilevel models, WASH effects were not statistically significant despite large PAFs. PAF is sensitive to exposure prevalence and crude prevalence ratio independent of multivariable significance, and wide adjusted odds-ratio intervals reflect small exposed sub-samples rather than a demonstrated null. The attenuation of the crude water–underweight association in the adjusted model was driven principally by wealth and region, indicating confounding by socioeconomic position and geography rather than an absence of association. High MOR values indicate pronounced unmeasured contextual heterogeneity, consistent with the Theil T evidence.

### Strengths and limitations

Strengths include the use of the 2022 GDHS with standardized WHO-compliant anthropometry and a validated sampling design, an analytical breadth that ties each measure to a stated objective, and a deliberate focus on young mothers that fills an evidence gap.

Several limitations apply. The cross-sectional design precludes causal inference; all associations, including the PAF estimates and regression coefficients, are descriptive and hypothesis-generating, and the large WASH PAFs should be read with the same caution as the confidence intervals. Because roughly 16% of eligible pairs were excluded and excluded pairs differed modestly on WASH access, missingness was not fully at random, and WASH-related distributions may be mildly affected by selection. Several regions have small subsamples (Greater Accra, Volta, Bono, Western North with *n* ≈ 20), producing wide confidence intervals; regional rankings and the Oti hotspot rest on imprecise estimates and are presented as exploratory, supported by a jackknife stability check and a sensitivity analysis excluding/merging small region. With only 16 areal units, both global and local spatial statistics have limited power and non-trivial Type I error risk. WASH variables are self-reported and binary and cannot capture service continuity, water quality, reliability or seasonality, dimensions particularly relevant given Ghana’s dry-season water access problems. The higher-education stratum (~5 children) is too small for stable inference and was removed as a reference for inequality ratios. The study was likely underpowered to detect WASH effects in multivariable models.

### Policy implications

The findings support subnational, equity-responsive targeting. The largest population-level attributable burden lies with improved water access, motivating accelerated rural water investment aligned with SDG 6.1 in the highest-burden northern and Volta-belt districts; sanitation coverage of 50.9% supports Community-Led Total Sanitation and hardware subsidies for the poorest households (SDG 6.2). The steep education gradient in stunting supports adolescent and girls’ education as a nutrition-sensitive investment. For young mothers specifically, integrated WASH, nutrition and reproductive-health programming within CHPS, reaching the 81% with media exposure, offers a high-coverage behavior-change channel. Because the evidence is cross-sectional, these are priorities for where to act, not guarantees of causal effect size.

## Conclusion

Stunting, wasting and underweight are prevalent among under-five children of young mothers in Ghana and are spatially heterogeneous, with the highest observed burden in the Volta belt and northern regions, though several of these regional estimates rest on small, imprecise subsamples. Global spatial autocorrelation was non-significant at regional level (all *p* ≥ 0.296); exploratory local analyses suggested possible clusters (Oti for underweight; Central and Bono as cold spots) that require confirmation at finer geographic scale. Improved water access constitutes the largest modifiable population-level attributable burden. All three outcomes are concentrated among the poorest and least-educated households; regional variation dominates wasting inequality, while region and wealth jointly drive underweight inequality. The multilevel models showed high discrimination, but this reflects combined measured and unmeasured community-level contributions rather than the sufficiency of the measured determinants. Because the design is cross-sectional, these conclusions describe where the burden lies and how much co-occurs with WASH deficits; they do not establish that WASH improvement would causally produce the estimated reductions.

## Data Availability

This study utilized fully anonymized, publicly available secondary data accessed through a formal online application to the DHS Program (https://dhsprogram.com) following registration and approval of a project proposal outlining the intended analyses.
